# Independent response modulation of visual cortical neurons by attentional and behavioral states

**DOI:** 10.1016/j.neuron.2022.08.028

**Published:** 2022-09-21

**Authors:** Takahiro Kanamori, Thomas D. Mrsic-Flogel

**Affiliations:** 1 Sainsbury Wellcome Centre for Neural Circuits and Behaviour, University College London, 25 Howland Street, London W1T 4JG, UK

## Abstract

Sensory processing is influenced by cognitive and behavioral states, but how these states interact to modulate responses of individual neurons is unknown. We trained mice in a visual discrimination task wherein they attended to different locations within a hemifield while running or sitting still, enabling us to examine how visual responses are modulated by spatial attention and running behavior. We found that spatial attention improved discrimination performance and strengthened visual responses of excitatory neurons in the primary visual cortex whose receptive fields overlapped with the attended location. Although individual neurons were modulated by both spatial attention and running, the magnitudes of these influences were not correlated. While running-dependent modulation was stable across days, attentional modulation was dynamic, influencing individual neurons to different degrees after repeated changes in attentional states. Thus, despite similar effects on neural responses, spatial attention and running act independently with different dynamics, implying separable mechanisms for their implementation.

## Introduction

Behaving animals dynamically integrate external sensory stimuli with internal brain states. Differences in brain state, such as variations in attention (Harris and Thiele, 2011; Speed et al., 2020), arousal (McGinley et al., 2015; Vinck et al., 2015), hunger (Burgess et al., 2016), and running (Bennett et al., 2013; Niell and Stryker, 2010; Vinck et al., 2015) can influence neuronal responses to sensory stimuli and animals’ behavior. For example, performance of primates in signal detection and discrimination tasks is better when they allocate their attention selectively to the task-relevant sensory stimuli (Bashinski and Bacharach, 1980; Downing, 1988; Handy et al., 1996; Hawkins et al., 1990). When mice are at intermediate arousal (as indicated by pupillometry), they have lower detection thresholds for sensory stimuli (Bennett et al., 2013; McGinley et al., 2015; Neske et al., 2019). The arousal of mice varies during running, resulting in different levels of task performance (McBride et al., 2019; McGinley et al., 2015).

Measurements of neural activity in the visual system suggest that attention and active behavior yield similar improvements in sensory processing (Dadarlat and Stryker, 2017; Harris and Thiele, 2011). In rodents, running increases sensory responses of neurons (Niell and Stryker, 2010) and desynchronizes neuronal populations in the primary visual cortex (V1) (Dipoppa et al., 2018; Ferguson and Cardin, 2020; Polack et al., 2013; Vinck et al., 2015), enhancing the visual processing and neural encoding of visual stimuli (Dadarlat and Stryker, 2017; Mineault et al., 2016). These effects resemble attentional modulation of cortical activity in primates (Chalk et al., 2010; Harris and Thiele, 2011; McAdams and Reid, 2005; Roelfsema et al., 1998). Recent rodent studies suggest a common circuit motif underlying these state-dependent influences: active behavioral states such as running can enhance cortical responses via a disinhibitory pathway involving different classes of interneurons (Fu et al., 2014; Gasselin et al., 2021; Jackson et al., 2016; Reimer et al., 2014; but see also Dipoppa et al., 2018; Pakan et al., 2016), a mechanism which has also been suggested to play a role in attentional modulation in V1 (Karnani et al., 2016; Zhang et al., 2014). Given these similarities, it has been assumed that spatial attention and running share common mechanisms to modulate the activity of cortical neurons. A recent study demonstrated that running modulated V1 neurons differently between two cohorts of mice that were trained on different visual tasks (spatial versus non-spatial tasks), suggesting the existence of some interaction between task engagement and running to modulate cortical activity (McBride et al., 2019). However, due to the lack of experimental paradigms in which both types of state-dependent modulation can be probed simultaneously in the same neuronal populations, it is unclear how individual neurons are similarly modulated by attentional and behavioral states. To address this issue, we designed a behavioral task for mice that allowed us to compare the influence of spatial attention and running on neural responses and found them to be uncorrelated with distinct dynamics.

## Results

### Visual discrimination task with spatial attention

**Figure 1:**
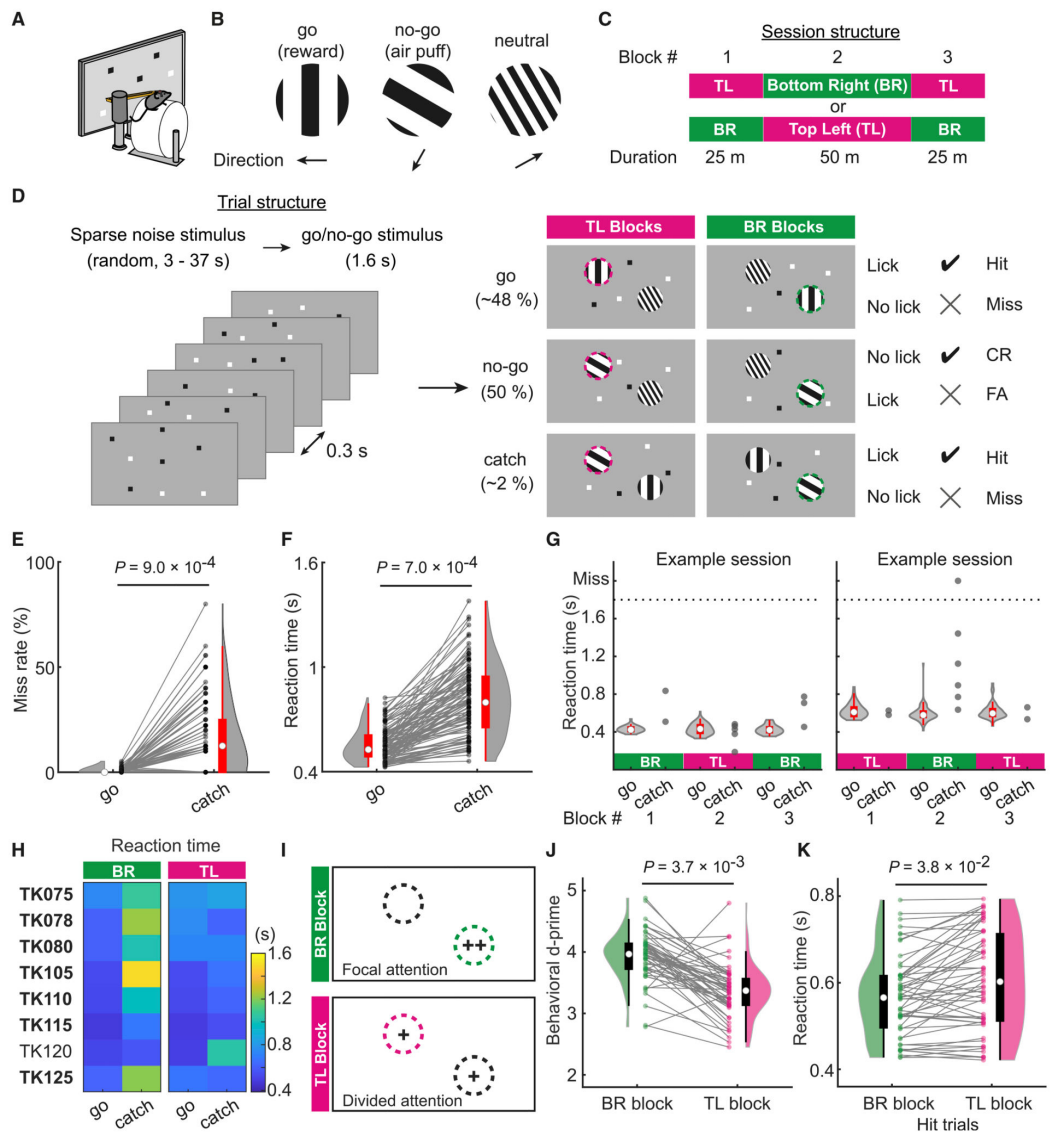
Visual discrimination task with spatial attention (A) A head-fixed mouse. Visual stimuli were presented in the left visual field. (B) Visual stimuli for the discrimination task. (C) Structure of three block sessions. (D) Structure of trials. After different sparse noise stimuli were shown every 0.3 s for a random duration, either a go or no-go stimulus was shown together with a neutral stimulus for 1.6 s. (E) Miss-rate in go and catch trials. Lines connect data from the same sessions. n = 111 sessions, 8 mice. (F) As in (E), reaction time. (G) Reaction time in example sessions. Gray dots, reaction time in all catch trials. (H) Mean reaction time in go and catch trials of BR and TL blocks for each mouse. (I) Spatial attention is focused or divided in the BR or TL block, respectively. Plus symbols, the location where mice direct spatial attention. (J) Performance in BR and TL blocks. n = 55 sessions, 7 mice. (K) As in (J), reaction time in hit trials. Catch trials were not included. Circles, boxes, and lines in violin plots indicate the median, 25%–75% quantiles, and the range of the data without outliers, respectively (E–G, J, and K). p values determined by hierarchical bootstrapping. See also Figures S1–S3.

We trained mice to discriminate two gratings of different orientations at one of two locations within the same hemifield (Figures 1A–1D). Each trial started with a delay period of random duration (95% confidence interval [CI], 2.8–37.0 s; see STAR Methods), during which sparse noise visual stimuli were shown every 0.3 s. Then, either a vertical (go) or an angled (no-go) grating stimulus was shown at one of the two locations (top-left, TL; or bottom-right, BR) for 1.6 s (Figures 1B and 1D). In response to the go stimulus, mice were trained to lick a spout for a liquid reward (hit trials, Hit). No reward was given when they failed to respond within 1.6 s (miss trials, Miss). When the no-go stimulus appeared, mice needed to withhold licking (correct rejection trials, CR), otherwise they were punished with an air puff (false-alarm trials, FA). Shown at the other location was a third type of stimulus (neutral stimulus) that had no relevance to receiving reward or punishment (Figures 1B and 1D). Therefore, mice needed to direct their attention to the location of go/no-go stimuli to decide whether to lick for reward.

To alternate the location to which mice direct their attention, each session was divided into either two (5 mice) or three blocks (4 mice), between which the locations of go/no-go stimuli switched (Figure 1C; Video S1; hereafter we denote the block where go/no-go stimuli are shown at the top-left or bottom-right location as the TL or BR block, respectively). Mice were free to run on a running wheel during the task and running speed was monitored together with the size and position of the pupil. No substantial differences were detected in these measures between TL and BR blocks (Figure S1). Mice performed marginally better during running (running speed ≥ 1 cm/s) than during stationary periods (running speed < 1 cm/s), showing significantly smaller miss and FA rates during running (Figure S2).

To test whether mice selectively attend to the locations at which they discriminate the visual stimuli, we introduced a small number of no-go trials in which the neutral stimulus was replaced with the go stimulus (Figure 1D, catch). Our rationale was that if mice spatially attended to the location where the go/no-go stimuli were shown, they would either fail to respond or respond with a delay to the go stimulus shown unexpectedly at the other location. Indeed, mice often did not respond (Figure 1E) or responded with a significantly longer reaction time (Figure 1F) to the go stimulus in catch trials. Consistent with primate studies in which response latency is linked to attention (Posner, 1980), these results suggest that mice can direct spatial attention even within the same hemifield.

Interestingly, each mouse had a bias in the locations to which it directed spatial attention (Figures 1H and S3). All but one animal either missed or reacted with delay in catch trials during BR blocks while responding at short latencies in catch trials during TL blocks (Figures 1G and 1H), suggesting that they focused attention selectively to the BR location during BR blocks but divided attention to both locations during TL blocks (Figure 1I). In humans, dividing attention has a substantial effect on performance in selective attention tasks, resulting in more errors and slower responses (Castiello and Umiltà, 1990; Eriksen and St James, 1986; Müller et al., 2003). Analyzing the mice that biased their focal attention to the BR location, we found that they showed similar phenotypes as humans: the task performance was better in BR blocks than in TL blocks, as quantified with behavioral d-prime (Figure 1J; see STAR Methods), and the reaction time in Hit trials was significantly shorter in BR blocks than in TL blocks (Figure 1K). One mouse showed focal attention to the TL location (Figure 1H, TK120 mouse), suggesting that it used a different strategy to solve the task. We therefore focused our analysis on the mice that biased their focal attention to the BR location.

### Spatial attention does not change the location or shape of V1 receptive fields

**Figure 2:**
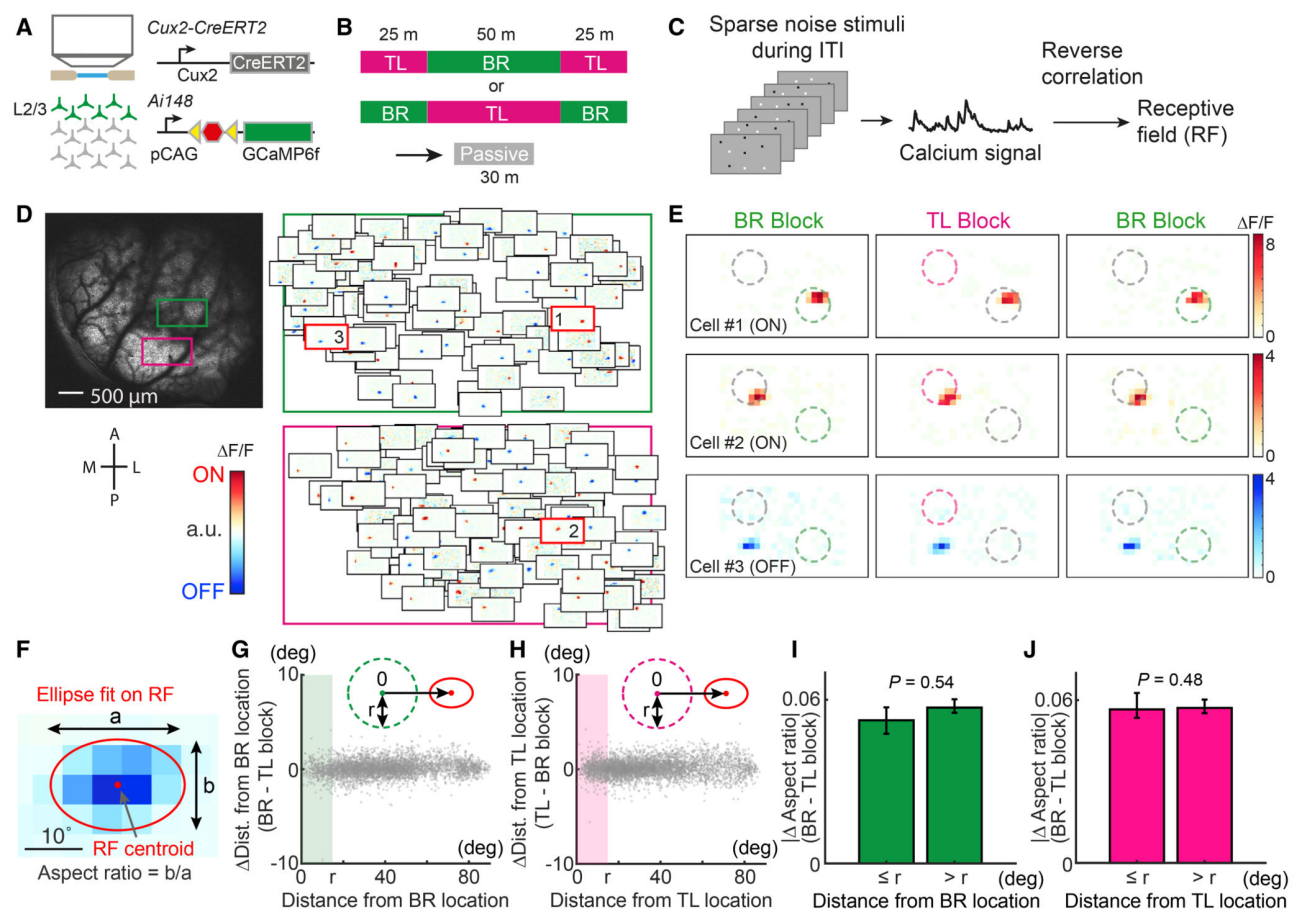
Spatial attention does not change the location or shape of V1 receptive fields (A) Calcium imaging was done in L2/3 excitatory neurons expressing GCaMP6f. (B) Structure of imaging sessions. (C) Receptive fields (RFs) were mapped by reverse-correlating calcium responses to the sparse noise stimuli, which were shown during inter-trial intervals (ITI). (D) Left, two simultaneously imaged fields-of-view in V1. Right, RFs of neurons in the two regions. The green and magenta rectangles covered regions that retinotopically corresponded to BR and TL locations, respectively. Red, ON field; blue, OFF field. (E) Three example neurons with different RF locations. RFs were mapped separately in the three blocks in a session. Their geometric locations within the fields-of-view are shown in (D). (F) A RF fitted with two-dimensional elliptical Gaussian. (G) Between-block difference in distance from BR location to RF centroids. Black arrow, measured distance (zero at the center of BR location); *r*, radius of circular go/no-go stimuli. The area of go/no-go stimuli at BR location is shaded in green. n = 4,853 RFs. (H) As in (G) for TL location. n = 5,307 RFs. (I) Between-block difference in RF shape for RFs near and distant from BR location. n (≤ *r*) = 443 RFs, n (> *r*) = 4,414 RFs. p values were determined by hierarchical bootstrapping. Error bars, 90% CI of bootstrapped mean. (J) As in (I) for TL location. n (≤ *r*) = 896 RFs, n (> *r*) = 4,414 RFs. See also Figure S4.

To examine how the sensory responses of V1 neurons were modulated by attention, we expressed the calcium indicator GCaMP6f (Chen et al., 2013) in excitatory neurons of supragranular and granular cortical layers using the Cux2-CreERT2/Ai148 transgenic mouse line (Figures 2A and S4). We chronically recorded calcium signals from L2/3 neurons in two V1 regions simultaneously, at least one of which retinotopically corresponded to the BR or TL locations, by using a large field-of-view two-photon microscope (Denk et al., 1990; Sofroniew et al., 2016) (Figures 2A and 2D, green and magenta rectangles). All recordings were carried out while animals performed the task for three blocks and passively viewed the sparse noise stimuli during an additional “passive block” (Figure 2B, 4 mice, 69 sessions).

Previous primate studies reported that spatial attention shifts receptive fields (RFs) of neurons in higher visual areas toward the attended location by shrinking their shape (Connor et al., 1997; Womelsdorf et al., 2006). To test whether neuronal responses in the mouse V1 change in a similar way, we first mapped RFs in BR and TL blocks separately by reverse-correlating the sparse noise stimuli with evoked responses (Figures 2C–2E; see STAR Methods). We fitted an ellipse on the RFs and examined whether the distances between the RF centroids and the BR/TL locations were different between BR and TL blocks (Figures 2E and 2F; see STAR Methods). We found that the between-block differences in the RF centroid distance to the BR or TL location were negligibly small (median: 0.076° or 0.093°, respectively) irrespective of the location of the RFs (Figures 2G and 2H). In addition, changes to the RF aspect ratio (ratio of the major and minor ellipse axes) were equally small for the RFs that were near and far from the BR/TL locations (Figures 2I and 2J). Thus, the retinotopic location and shape of RFs were stable irrespective of where mice attended spatially.

### Attentional modulation of visual responses in local L2/3 excitatory neurons

**Figure 3:**
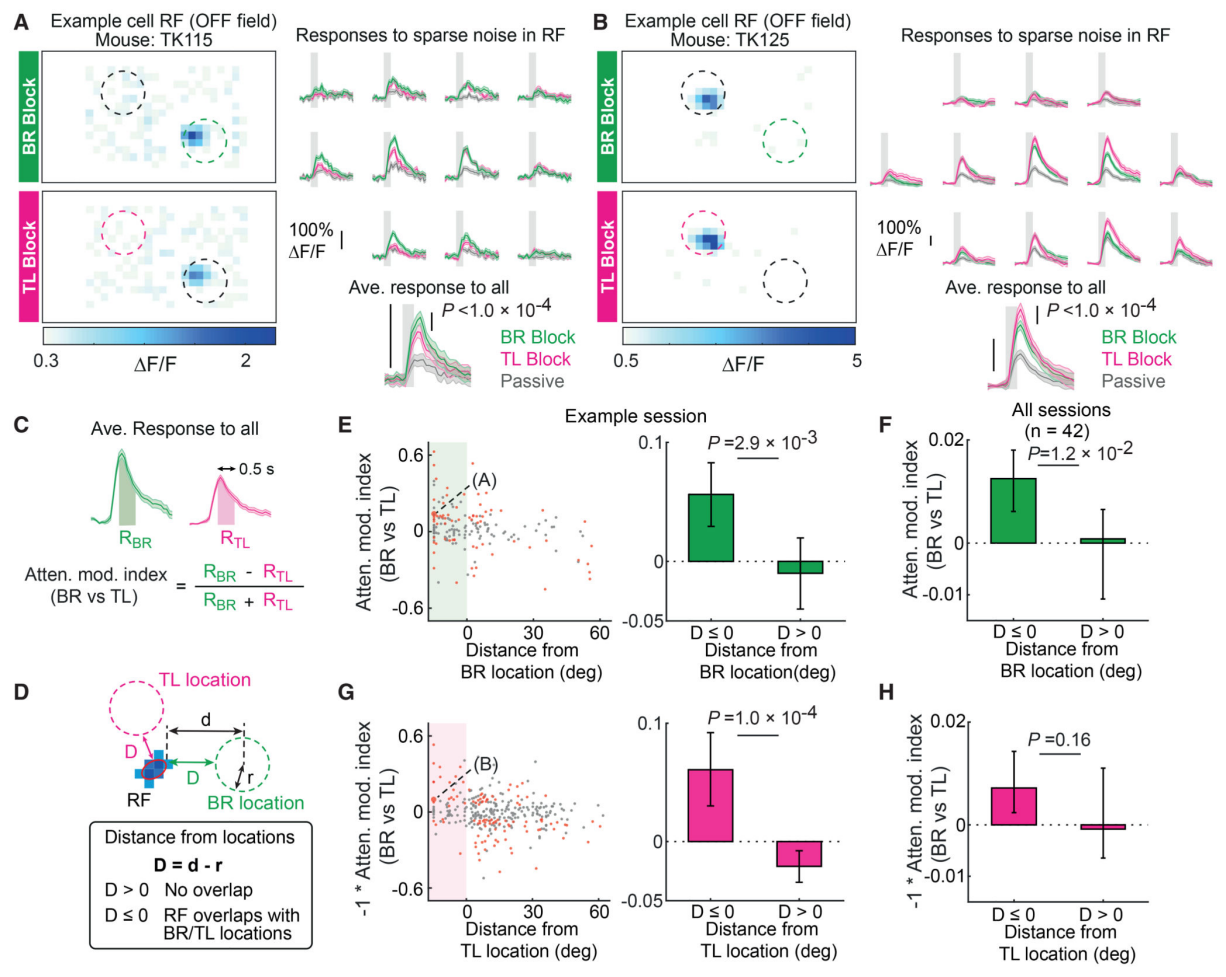
Stronger visual responses of L2/3 excitatory neurons at attended locations (A) An example cell with attentional modulation. Left, RFs mapped separately in BR and TL blocks. Right, calcium responses to each sparse noise stimulus within the RF in BR, TL, and passive blocks, and those averaged across all sparse noise stimuli for each block type. Shaded area, 68% CI of bootstrap estimate of the mean. p values were from bootstrap test and adjusted by the Benjamini-Hochberg procedure (FDR = 0.05). (B) As in (A), another example cell. (C) Attentional modulation index. (D) Calculation of distance between an RF and BR/TL location. (E) Response modulation by spatial attention to BR location in an example session. Left, scatterplot showing cells whose responses to the sparse noise stimuli were significantly different (red) or not different (gray) between BR and TL blocks. The example neuron in (A) is indicated with a bigger dot. Right, attentional modulation of neurons with RFs at or away from BR location. p value determined by bootstrap test. n (≤ *r*) = 95 RFs, n (> *r*) = 77 RFs. Error bars, 90% CI of bootstrapped mean. (F) Response modulation by spatial attention to BR location in all sessions. p value determined by hierarchical bootstrapping. n (≤ *r*) = 1,841 RFs, n (> *r*) = 5,368 RFs. Error bars, 90% CI of bootstrapped mean. (G) As in (E), spatial attention to TL location. The sign of the index was reversed to assess attentional modulation in TL blocks relative to BR blocks. n (≤ *r*)= 63 RFs, n (> *r*) = 259 RFs. (H) As in (F), spatial attention to TL location. n (≤ *r*) = 3,875 RFs, n (> *r*) = 5,368 RFs. 42 sessions from 3 mice. See also Figures S5 and S6.

Neurons in the visual cortex have been found to respond more strongly to stimuli within their RFs when animals attend to those stimuli as opposed to when they attend elsewhere (Carrasco, 2011; Speed and Haider, 2021; Speed et al., 2020; Treue, 2001). To test whether neurons in the mouse V1 are modulated in a similar way in our spatial attention task, we investigated the relationship between the magnitude of attentional modulation and the location of RFs relative to the BR and TL locations (Figures 3D–3H). To probe the response modulation by spatial attention, we measured neuronal responses to the sparse noise visual stimuli that were shown during the inter-trial interval delay periods (Figure 1D), which were irrelevant to the task and induced neither overt movements nor changes in arousal level (Figure S5). This task design was chosen to minimize the confounding effects of body movement, which strongly affects neural activity in many brain areas, including V1 (Musall et al., 2019; Niell and Stryker, 2010; Salkoff et al., 2020; Stringer et al., 2019). The effect of attentional modulation was quantified by comparing the magnitude of averaged responses to all sparse noise stimuli within the RF between BR and TL blocks (Figures 3A–3C, attentional modulation index). The overlap of RFs with BR/TL locations was defined by the minimum distance (*D*) between the fitted ellipse to RFs and BR/TL locations (Figure 3D, with overlap if *D* ≤ 0).

By quantifying response modulation in BR blocks relative to TL blocks, we first found that neurons whose RFs overlapped with the BR location were more strongly positively modulated than those whose RFs were distant from it (Figures 3A, 3E, and 3F). This indicates that response modulation by spatial attention to BR location is stronger when mice focused their spatial attention than when they attend to the same location by dividing it, which is consistent with studies in primates showing more pronounced attentional modulation by focused than divided attention (Mayo and Maunsell, 2016; McMains and Somers, 2005; Müller et al., 2003). Next, we reasoned that because TL location is attended only in TL blocks when mice divided their attention to both locations (Figure 1I), neurons whose RFs overlapped with TL stimuli would show stronger responses in TL blocks than in BR blocks. This was indeed the case in some sessions, in which significantly larger responses were seen in neurons with RFs at the TL location than those with distant RF centroids (Figures 3B and 3G; note that the sign of the attentional modulation index was reversed to assess attentional modulation in TL blocks relative to BR blocks). A similar trend was observed when all sessions were pooled together, although the difference did not reach the significance criterion (Figure 3H).

To gain more insight into how neurons were modulated by focal and divided attention, we compared visual responses in BR and TL blocks against responses to the same stimuli passively viewed outside of the task context. We expected that during BR blocks, when attention is focused, neurons with RFs at the BR location but not at the TL location would be more strongly modulated than neurons whose RFs have no overlap with either location (Figure S6C, compare top and bottom). In contrast, during TL blocks, when attention is divided, neurons with RFs at both the BR and the TL locations should show stronger modulation than neurons with RFs outside of these locations (Figure S6C, compare middle and bottom). Indeed, when BR blocks were compared against the passive condition (Figure S6A), neurons with RFs at the BR location but not at the TL location showed significantly stronger attentional modulation than those with RFs distant from both locations (Figure S6D). When TL blocks were compared against the passive condition (Figure S6B), larger attentional modulation was observed for both neurons with RFs overlapping with the TL and the BR location than those with RFs distant from both locations (Figure S6E). Together, these results suggest that the local populations of V1 neurons whose RFs overlap with spatially attended visual field locations boost their responses to stimuli at those locations.

### Independent modulation of individual neurons by spatial attention and running

**Figure 4:**
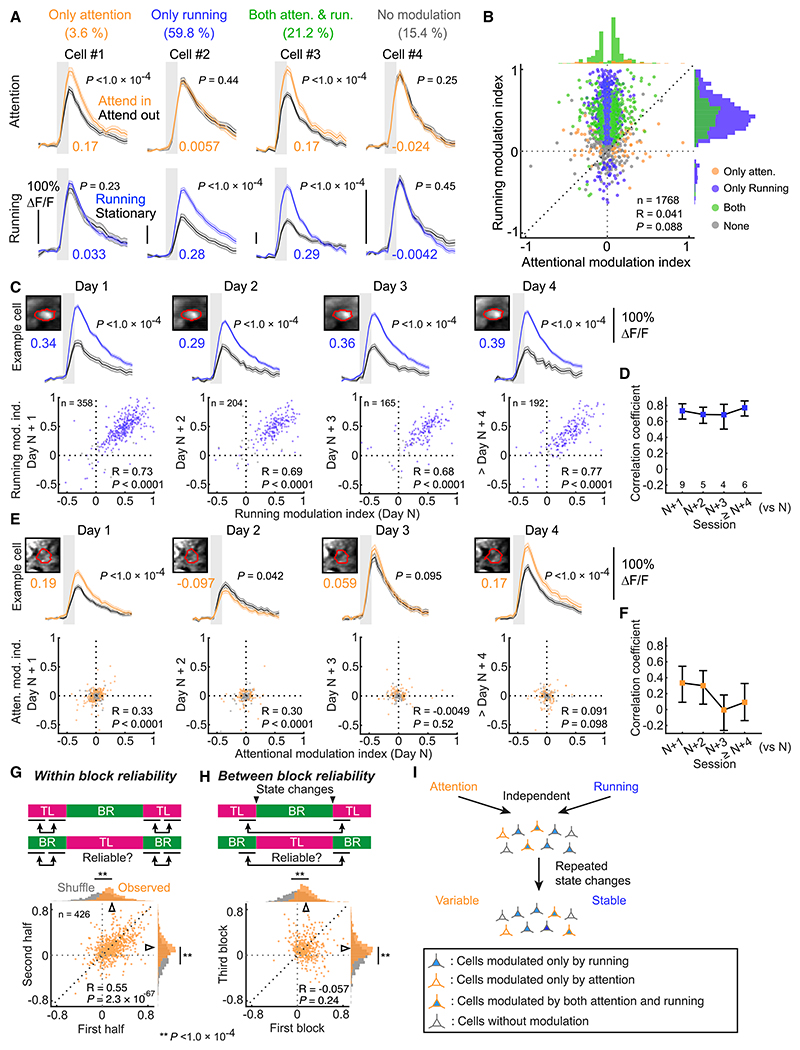
Response modulation by spatial attention and running is independent and different in reliability (A) Example cells showing different response modulation patterns. Upper traces, visual responses when mice attend in or out of the RF location. Lower traces, visual responses when mice are running or stationary. The values under the traces are modulation indices. (B) Scatterplot showing no correlation between attentional and running modulation indices. (C) Top, an example cell showing stable running-dependent modulation across days. Corresponding cell images and regions of interest (red) are shown for each day. Values below images are modulation indices. Bottom, scatterplots comparing running modulation index between day N and consecutive days. Data from more than 4 days after the first day were pooled together. (D) Correlation of running modulation index across days. Error bars, 90% CI of bootstrapped mean. The numbers of session pairs are indicated at the bottom. (E) As in (C), attentional modulation. (F) As in (D), attentional modulation. (G) Within-block reliability of attentional modulation. The scatterplot compares the modulation indices between the first and second half of the same blocks. (H) Between-block reliability of attentional modulation. Attentional modulation indices were calculated separately for the first and third blocks and compared in the scatterplot. Same neurons as in (G). p values in (G) and (H) were determined by a randomization test. (I) Summary. Two types of state-dependent response modulation are imposed on single neurons independently. After repeated state changes, single neurons are either variably or stably modulated by spatial attention and running, respectively. p values of all example neurons were determined by bootstrap test. All calcium response traces are mean response to all sparse noise stimuli within RFs. See also Figures S7–S10.

We next addressed how individual neurons were modulated by global changes in behavioral state, such as during running. We considered two possibilities. First, as implied by previous studies in mice (Dadarlat and Stryker, 2017; Mineault et al., 2016), attention- and running-dependent modulation of neural responses might engage similar mechanisms, such that neurons modulated by attention may also be more likely to be modulated by the active behavioral state. Alternatively, individual neurons could be modulated independently such that the magnitude of modulation by one state has no relation to that imposed by the other. To differentiate between these possibilities, we first characterized how visual responses of V1 neurons were modulated by running. After dividing each imaging session into running (running speed ≥ 1 cm/s) and stationary (running speed < 1 cm/s) epochs, we assessed response modulation by running by calculating a running modulation index, the difference of the average responses to all sparse noise stimuli within the neuron’s RF between running and stationary periods divided by their sum (see STAR Methods). Consistent with previous reports (Bennett et al., 2013; Dipoppa et al., 2018; Niell and Stryker, 2010; Polack et al., 2013), the majority of neurons showed stronger visual responses when mice were running than when stationary (Figures 4A and 4B, see vertical axis). We then examined the relationship between modulation by spatial attention and running for individual neurons. Since attentional modulation for the BR location was more robust than for the TL location (Figures 3F and 3H), we only analyzed neurons whose RFs were at the BR location (Figure 4B, horizontal axis: the same index as in Figure 3C). A substantial number of neurons (21.2%) were significantly modulated by both spatial attention and running (Figure 4A, example cell 3), indicating that the activity of individual neurons can be influenced by both states. However, there was almost no correlation between the strength of modulation by spatial attention and by running (Figure 4B, R = 0.041, p = 0.088), supporting the idea that the two states modulate neural responses separately.

Running also modulates baseline activity of V1 neurons at least in part by inducing responses at running onsets (Dipoppa et al., 2018; Leinweber et al., 2017; Niell and Stryker, 2010; Polack et al., 2013; Saleem et al., 2013; Vinck et al., 2015). As reported previously (Dipoppa et al., 2018), modulation of baseline activity was broadly distributed in L2/3 V1 excitatory neurons, showing both positive and negative running-onset activities (Figure S7A), and was not correlated with that of visual responses (Figure S7B). Again, we found that there was no correlation between the magnitude of running-onset activity and the attentional modulation index (Figure S7C). Taken together, we conclude that spatial attention and running have independent influences on the neural activity of the individual V1 neurons.

### Running-dependent modulation is stable, while attentional modulation is dynamic over time

Little is known about how reliably individual neurons are modulated in response to repeated state changes. We tracked the modulation of neural activity by spatial attention and running across sessions in which mice showed a similar spatial attention phenotype (see STAR Methods). Consistent with a previous study (Ranson, 2017), the influence of running on visual responses was highly similar across days (Figures 4C and 4D). Likewise, the magnitude of running-onset activity was highly stable (Figure S8). In contrast, response modulation by spatial attention was much less stable over time (Figures 4E and 4F). This difference was not due to the relatively smaller magnitude of attentional modulation compared with modulation by running (Figure S9). Moreover, across-day variability of gaze direction is unlikely to explain the day-to-day differences in attentional modulation: overall shifts in RFs of recorded neurons between sessions (95% CI of RFs shift relative to a reference session: azimuth, [posterior, anterior] = [3.02°, 1.44°]; elevation, [up, down] = [1.08°, 2.11°]; 30 sessions) were much smaller than the average V1 neuron RF size (mean major/minor RF axes, 21.6°/14.8°; 11,018 RFs).

To more explicitly test whether repeated changes in attentional state trigger this variability of attentional modulation, we assessed the stability of attentional modulation over shorter time periods. We hypothesized that attentional modulation should be stable within a block in which mice were continuously in the same attentional state, and that attentional modulation may become more variable between the first and third blocks, in which mice discriminated stimuli at the same location, but separated by a block in which mice attended to another location, involving repeated changes in attentional state. We first examined the reliability of attentional modulation within a block for neurons that were significantly positively modulated by spatial attention. The attentional modulation indices in the first and second half of a block (see STAR Methods) were strongly correlated (Figure 4G), indicating that attentional modulation is robust and stable when mice remain attentive to the same spatial location. In contrast, attentional modulation indices were not correlated between the first and the third blocks (Figure 4H), even though mice attended to the same location and V1 responses were significantly modulated by spatial attention across the population in both blocks (Figure 4H, open arrowheads). For comparison, response modulation by running was highly stable within and across blocks during a session (Figure S10). Taken together, these results suggest that attentional modulation is reliable if mice remain in a continuous attentional state, but single neurons are modulated with different magnitudes after shifts in attentional state. Thus, spatial attention and running modulate neuronal activity in distinct dynamics, providing further evidence that these two types of state-dependent modulation are independently imposed on individual neurons (Figure 4I).

## Discussion

By introducing a new spatial attention task in mice, we found that the response modulation associated with spatial attention was independent of the response modulation associated with an active behavioral state such as running.

What are the circuit mechanisms underlying the modulation of V1 neurons by spatial attention and running? Previous primate studies have indicated that spatial attention engages long-range networks involving the fronto-parietal cortex, the superior colliculus, the basal ganglia (Krauzlis et al., 2013; Noudoost et al., 2010; Womelsdorf and Everling, 2015), and neuromodulatory systems (Harris and Thiele, 2011; Thiele and Bellgrove, 2018). Recent mouse studies demonstrated the involvement of the superior colliculus and the dorsomedial striatum in spatial attention tasks (Hu and Dan, 2022; Wang and Krauzlis, 2020; Wang et al., 2022), suggesting similar mechanisms in mice and primates. Running-dependent modulation likely involves neuromodulatory pathways. Both noradrenergic and cholinergic axon terminals in V1 are activated in active behavioral states (Reimer et al., 2016). Noradrenergic signaling contributes to neuronal activities of L2/3 V1 excitatory neurons at running onset (Polack et al., 2013). The ongoing modulation of visual responses by running is likely mediated by the cholinergic system because optogenetic activation of cholinergic fibers increases the response gain of V1 neurons (Pinto et al., 2013; but see also Chen et al., 2015) in a similar way as running does (Niell and Stryker, 2010). In addition, running-onset activity in V1 neurons involves inputs from the anterior cingulate cortex (Leinweber et al., 2017), which are also thought to contribute to the attentional modulation of visual responses (Zhang et al., 2014). Therefore, the two types of modulation by spatial attention and running likely rely on partly overlapping circuit components.

However, how these different sources of input are processed in the local circuit to influence individual cortical neurons is still not well understood. Our results raise the possibility that modulation by spatial attention and running is imposed independently at the level of individual L2/3 excitatory neurons in V1. While previous studies suggested similarities in neural activity modulation in V1 of selectively attending monkeys and actively behaving mice (Chalk et al., 2010; Dipoppa et al., 2018; Harris and Thiele, 2011; McAdams and Reid, 2005; McGinley et al., 2015; Niell and Stryker, 2010; Roelfsema et al., 1998; Speed et al., 2020; Vinck et al., 2015), we propose that such similar effects are caused by separable neural circuit mechanisms engaged by different behavioral and cognitive states to modulate the activity of individual cortical neurons. Considering the likely overlap in the top-down circuitry as discussed above, our findings imply that there must be some way of parallelizing the influence of the two state-dependent modulatory mechanisms on the activities of layer 2/3 neurons in the visual cortex.

One caveat when interpreting our results is that response modulation by spatial attention was relatively small compared with that by running, which could have potentially hindered the detection of any interaction between the two types of modulation. There are several possible explanations for the small effects of attentional modulation in our experimental conditions. First, attentional modulation is typically weaker in V1 than in higher visual areas in primates (Klein et al., 2014; Treue, 2001). Second, any noise inherent to calcium imaging may reduce sensitivity to detecting such small changes in the firing rate. Finally, L2/3 excitatory neurons might not be the population that is most potently modulated by spatial attention, although they are reported to receive stronger long-range inputs from the anterior cingulate cortex than L5 neurons in the mouse V1 (Leinweber et al., 2017). Notwithstanding, the separability of attention- and running-dependent influences was clearly apparent when only considering neurons that were modulated by attentional modulation to a similar level to that imposed by running (Figure S9).

A considerable number of neurons showed significantly different visual responses between BR and TL blocks, irrespective of their RF location (Figures 3E and 3G), red dots in non-shaded areas). There are several possibilities for such RF location-independent response modulation. First, in our visual stimulation paradigm, wherein multiple sparse noise stimuli were given at the same time, neurons with RFs further away from the attended location could be additionally driven by attentionally modulated neurons with RFs at the attended location if they share a preference for similar visual features (Cossell et al., 2015; Iacaruso et al., 2017). Thus, spatial attention may have some indirect modulatory effect on neurons whose RFs are displaced from the attended location. Second, we speculate that this modulation could be explained by attention to visual features of go/no-go stimuli, as feature-based attention is reported to modulate the visual responses of neurons that share the preference to attended visual features irrespective of their RF locations in primates (Saenz et al., 2002).

Our study adds to the emerging evidence that mice are capable of deploying spatial attention (Hu and Dan, 2022; Speed et al., 2020; Wang and Krauzlis, 2018). We demonstrate that mice can allocate attention to different locations, even within the same hemifield. Interestingly, each mouse had a “preferred” location on which to focus their spatial attention. Although it is currently unclear why mice showed such a bias in spatial attention, visual perception of the mouse might be spatially asymmetric within the visual field. In addition, our mice performed the spatial attention task slightly better during running than during stationary periods. This is consistent with the observation that running improves the coding accuracy of the visual cortex in mice (Christensen and Pillow, 2022; Dadarlat and Stryker, 2017) but not with a recent study reporting the negative effect of running on the performance of a spatial-visual task (McBride et al., 2019). One possible explanation for this discrepancy is that mice in the two studies can be in essentially different states. Indeed, in the McBride et al. study, visual responses of L2/3 neurons are negatively modulated when mice are engaged in their spatial-visual task.

The magnitude of attentional modulation in individual neurons is variable after repeated changes in attentional states, but it remains similar if mice remain in the same attentional state (Figure 4). Although we cannot rule out that this increased variability may arise from any changes in visual responses in relation to lapsed time in a session (see STAR Methods for how we controlled for the time-dependent response changes), it suggests that the patterns of functional influence of top-down inputs on the V1 neurons may be overlapping but not identical after repeated changes in attentional states. In support of this hypothesis, the same task strategies have been shown to be represented by different population activity patterns in the rat prefrontal cortex after multiple strategy switching within the same session (Malagon-Vina et al., 2018). Thus, spatial attention is inherently a dynamic process that appears to target different cohorts of neurons according to the feature that is being prioritized. This is in stark contrast to stable modulation by running, which may be intrinsically conferred by the expression of neuromodulatory receptors (Bugeon et al., 2022). By taking advantage of an extensive repertoire of mouse genetic tools, our paradigm can provide an avenue for understanding the circuit mechanisms not only for separating the influence of spatial attention from that of running but also for both focal and divided attention.

## Star⋆Methods

## Key Resources Table

**Table:** 

REAGENT or RESOURCE	SOURCE	IDENTIFIER
Chemicals, peptides, and recombinant proteins		
RNAscope Target Probe GFP	Advanced Cell Diagnostics	400281-C2
RNAscope Target Probe Gad2	Advanced Cell Diagnostics	439371-C3
Tamoxifen	Sigma-Aldrich	T5648-1G
Critical commercial assays		
RNAscope Fluorescent Multiplex Reagent Kit	Advanced Cell Diagnostics	320850
Experimental models: Organisms/strains		
Mouse: Cux2-CreERT2	The Jackson Laboratory	RRID: IMSR_JAX:012243
Mouse: Ai148: B6.Cg-Igs7^tm148.1(tetO-GCaMP6f,CAG-tTA2)Hze^ /J	The Jackson Laboratory	RRID: IMSR_JAX:030328
Software and algorithms		
ScanImage	Vidrio Technology	https://vidriotechnologies.com/scanimage/
MATLAB, 2018a, 2019a	MathWorks	https://uk.mathworks.com/products/matlab.html
LabView	National Instruments	https://www.ni.com/en-gb/shop/labview.html
Custom program for visual stimulation	Voitov and Mrsic-Flogel, 2022	https://github.com/Ivan-Voitov/ViZi
Suite2p	Pachitariu et al., 2017	https://github.com/MouseLand/suite2p
CellPose	Stringer et al., 2021	https://github.com/MouseLand/cellpose
CellReg	Sheintuch et al., 2017	https://github.com/zivlab/CellReg
ImageJ	NIH	https://imagej.nih.gov/ij
Custom code for experiments	This paper	https://doi.org/10.5281/zenodo.6640207
Custom code for data analysis with processed data	This paper	https://doi.org/10.5281/zenodo.6640217

## Resource Availability

### Lead contact

Further information and requests for resources and reagents should be directed to and will be fulfilled by the lead contact, Thomas D. Mrsic-Flogel (t.mrsic-flogel@ucl.ac.uk).

## Materials availability

The materials used in this study are commercially available.

## EXperimental Model And Subject Details

All procedures were carried out in accordance with the ethical guidelines licensed by the United Kingdom Home Office. In this study, we used a total of 8 mice, including male (*n*=5) and female (*n*=3) mice (90-238 days old). All mice were Cux2-CreERT2/Ai148 double heterozygous transgenic lines (JAX 012243 and JAX 030328, Jackson Laboratory).

## Method Details

### Surgical procedures

Prior to surgery, dexamethasone (2 - 3 mg per kg) and carprofen (5 mg per kg) were injected subcutaneously. All surgeries were done under isoflurane anesthesia (1 - 4%). A custom-designed metal headplate was implanted using dental cement (Super-Bond C&B, Sun Medical). A 5 mm craniotomy was made over the V1 of the right hemisphere and a 300 μm thick 5 mm diameter glass window (Matsunami) was implanted with cyanoacrylate glue (Pattex).

### Behavioral setup

Behavioral setups consisted of a styrofoam wheel, a visual stimulation display monitor, a reward delivery spout, an air puff tube, and two cameras for recording the eye movement. Mice were head-fixed and placed on a styrofoam wheel. The rotation of the wheel was recorded using a rotary encoder (05.2400.1122.1000, Kübler). Visual stimuli were displayed on a computer monitor (U2715H, Dell; 60 Hz refresh rate), placed 22.5 cm away from the left eye of mice and oriented at 20°relative to midline. The precise timing of visual stimulus onsets was recorded with a photodiode (Thorlabs) attached to the monitor. The reward delivery spout was placed in front of the animal and licks were detected with a piezoelectric diaphragm sensor (7BB-12-9, Murata) placed under the spout. In response to lick detection, a drop of rewarding milk (10 % soymilk in 10% sucrose water or 10% Ensure nutrition shake) was delivered by opening a solenoid pinch valve (161P011, NResearch). The air puff tube was placed in front of the right cheek and an air puff was given by opening a two-way electronic valve (EV-2-12, Clippard). Two cameras (22BUC03, ImagingSource) were positioned to take the side and low-angle views of the left eye at 30 Hz. The recording of the encoder, presentation of visual stimuli, opening of the valves, and camera recordings were controlled by custom written software in Matlab and LabView, which also recorded the frame trigger signal during two-photon imaging. Behavioral data were acquired using a PCIe 6321 acquisition card (National Instruments).

### Visual stimuli

We used visual stimuli that are similar to the “Locally Sparse Noise” in Allen Brain Observatory (de Vries et al., 2020). The sparse noise stimuli were black (0.14 cd/m^2^) and white (22.7 cd/m^2^) 5°squares appearing at 14 (elevation) ×22 (azimuth) locations in the gray back-ground (10.4 cd/m^2^). Multiple (7.7 on average; 95% confidence interval [CI], 6 to 10) sparse noise stimuli, each of which was spatially separated at least by 25°, were simultaneously shown on the monitor. Different patterns of the sparse noise stimuli were shown every 0.3 s, and those at the same location were temporally separated at least by 3 s (mean interval, 12.9 s; 95% CI, 3.6 to 39.0 s). Different sequences of sparse noise stimuli were shown in each session by randomly selecting from the ten pre-defined sequences. For the visual discrimination task, two circular patches of sinusoidal gratings (30°diameter) were shown at top-left (TL, azimuth 93°, elevation 18°) and bottom-right (BR, azimuth 36°, elevation -18°) locations together with the sparse noise stimuli for 1.6 s. The go stimulus was a vertical grating with the spatial frequency of 0.08 cycles per degree (cpd) moving from right to left (i.e., anterior to posterior); the no-go stimulus was an angled grating with 0.08 cpd moving in the direction that was rotated by 60°relative to the go stimulus; and the neutral stimulus was an angled grating with 0.16 cpd moving in the direction that was rotated by 150°relative to the go stimulus. In some sessions, grating stimuli of lower contrast were used to make the task more difficult. All visual stimuli were generated with a custom written UDP-interfaced visual stimulus presentation program (Voitov and Mrsic-Flogel, 2022).

### Behavioral task

A session had 377 ± 19 trials (mean ± SD). Each trial started with a delay period where different patterns of the sparse noise stimuli were shown every 0.3 s. The duration of the delay period was randomly selected from the exponential decay distribution with its mean 9 s. The lower bound was set to 3 s by shifting the whole distribution by that value, resulting in 11.7 s mean duration (95% CI, 2.8 to 37.0 s). After the delay period, two circular drifting grating stimuli, the go (or no-go) and neutral stimuli, were shown for 1.6 s together with the sparse noise stimuli still changing every 0.3 s. The location of go/no-go stimuli was blocked and switched once or twice in the two or three block sessions, respectively. Mice were trained to discriminate between go and no-go stimuli shown at one location while ignoring the neutral stimulus shown at the other location. In go trials, if mice licked a spout within 1.6 s after the onset, they were rewarded with a drop of rewarding milk (hit trials); otherwise, no reward was given (miss trials). In no-go trials, if mice made lick responses, they received an air puff to the cheek (false-alarm trials); but if they withhold licking during the whole 1.6 s of visual stimulation, they received no air puff (correct rejection trials). In a small number of trials (2.3 ± 0.3 % of trials, mean ± SD), the neutral stimulus of no-go trials was replaced with the go stimulus (catch trials). In catch trials, if mice reacted to the go stimulus at the unexpected location, they were rewarded just as in go trials. To avoid having several catch trials in a short time period, each catch trial was separated by at least ten rewarded trials. After the switch of blocks, no catch trials were introduced until animals got rewarded for ten trials in the new blocks.

### Behavioral training

Four to seven days after surgery, food-restriction started and body weights were maintained at the 85 % level. Before starting any training procedures, the animals were handled and received the rewarding milk on the experimenter’s hands for at least two sessions until they got used to the experimenter. Mice were trained in three steps.

Step 1. *Full screen go/no-go task*. Mice were head-fixed and put on the running wheel either in the training box or at the two-photon imaging setup. Shown on the monitor were patterns of triangles with different sizes and color (black and white) on the gray background whose movement was coupled to the rotation of the running wheel (closed-loop). Once animals started constantly running on the wheel, the vertical grating was displayed fully on the screen and a drop of the rewarding milk was given immediately and automatically at variable distances in the “virtual” environment. Animals usually formed an association between the vertical grating and reward quickly, and after this point the reward was given just after they licked in response to the vertical grating. Once mice stopped licking the spout constantly out of the vertical grating periods but started making lick responses selectively to the vertical grating without any miss, we stopped showing the triangle pattern and introduced the angled no-go stimulus. After mice started withholding licking in response to the no-go stimulus, the movement of the grating stimuli was decoupled from the rotation of the running wheel (open-loop). It usually took 3-7 days to complete this step.

Step 2. *go/no-go task with circular and smaller stimuli*. We started using the go/no-go stimuli with a circular shape and changed their sizes and locations gradually toward the final stimulus configurations. Once mice started successfully discriminating the go and no-go stimuli at both BR and TL locations without missing, the neutral stimulus was introduced. To facilitate mice to dissociate the neutral stimulus from either getting reward or receiving an air puff, the neutral stimulus was occasionally shown alone during the inter-trial interval until they stopped making lick responses to it. It usually took 3-7 weeks for mice to complete this step.

Step 3. *Introduction of the sparse noise stimuli*. Finally, the sparse noise stimuli were introduced during the inter-trial interval and then also together with the go/no-go stimuli. It usually took 5-8 weeks in total for mice to become expert in the final spatial attention task.

### Two-photon calcium imaging

To examine how sensory responses of V1 neurons were modulated by attention, we expressed the calcium indicator GCaMP6f (Chen et al., 2013) in excitatory neurons of supragranular and granular cortical layers using the Cux2-CreERT2/Ai148 transgenic mouse line. GCaMP6f expression was induced by intraperitoneally injecting tamoxifen (10 mg ml^-1^) for three times, every other day, at least 10 days before starting calcium imaging. We imaged the calcium dynamics in layer 2/3 excitatory neurons in V1 using a mesoscopic two-photon microscope (Multiphoton Mesoscope, Thorlabs) at 930 nm excitation wavelength (Mai Tai, SpectraPhysics). Two fields of view (~615 μm x 1166 μm) covered the areas in V1 that corresponded to at least one of the TL and BR locations. Images were acquired at 12.85 Hz with the software ScanImage (Vidrio Technology). The visual stimulation display was turned on only during the turnaround phase of the resonant mirror, during which images were not acquired. Imaging was done chronically for 3-7 days for each field of view. To minimize day-to-day differences in the eye angle relative to the display, one of the arms of the headplate was inserted into a plastic hole attached to the fixed head post. The signal from the galvanometer was recorded and used for synchronization between the calcium imaging frames and task related data (e.g., behavior data and visual stimuli onsets).

### *In situ* hybridization

Mice were deeply anesthetized with pentobarbital (80 mg/kg) and decapitated. Brains were quickly removed, embedded in cryoembedding medium (OCT), and stored at -80°C until use. Brains were sectioned on a cryostat (OTF 5000, Bright Instruments) into 15 μm sections. In situ hybridization (RNAscope) was conducted as described in the Advanced Cell Diagnostics (ACD) RNAscope Fluorescent Multiplex Assay manual. Probes for detecting GCaMP6 (cat # 400281-C2) and Gad2 (cat # 439371-C3) were commercially available from ACD. Images were taken with the slide scanner (Zeiss Axio ScanZ1, Carl Zeiss).

### Quantification And Statistical Analysis

### Behavioral data analysis

*Reaction time* Reaction time was calculated as the duration from the digital output signal triggered at the onset of go stimulus to the first lick after the onset. We excluded the following trials from analysis: 1) trials in which the lick sensor was triggered within 200 ms before the onset of go stimulus because mice often kept hitting the spout with forepaws in most of such trials, and 2) trials in which reaction time was too short (cut-off under ~160 ms). The reaction times of Miss trials were set to 1.6 s when calculating the mean reaction time.

#### Performance

Task performance was quantified with the behavioral d-prime as described previously (Poort et al., 2015). Briefly, it is the difference between normal inverse cumulative distribution function of Hit rate and that of False alarm rate (FA rate).

$$d^{\prime }={\text{Φ}}^{-1}(\;Hit\;rate\;)-{\text{Φ}}^{-1}(\;FA\;rate\;)$$

When the Hit rate was 100% (i.e., no misses occurred), it was given as (Stanislaw and Todorov, 1999):

$$\;Hit\;rate\;=\frac{\;Number\;of\;Hit\;trials\;-0.5}{\;Number\;of\;Hit\;trials\;}$$

#### Pupil size and position

Movies from the eye cameras were processed with the Matlab VideoReader function. After cropping the region around the eye, the pupil was defined by k-means clustering and an ellipse was fitted on the resulting binary images by using a Matlab function fitellipse.m (Brown, 2022). The area, position, and angle of pupil were calculated from the fitted ellipses. The deviations of the pupil position relative to its median position during the sparse noise period were calculated as angles in degrees for both azimuth and elevation. Video frames in which mice were not staring at the monitor (e.g., eye closure and grooming events) were identified as those whose 2-D correlation coefficient with the average image was smaller than the median correlation coefficient by more than 5.5 standard deviation and corresponding two-photon imaging frames were excluded from analysis.

#### Running speed

Running speed was calculated by converting the rotary encoder signal and filtered over 500 ms by the Savitzky-Golay filtering.

### Neural data analysis

#### Neural data processing

Two-photon imaging frames were registered and segmented using suite2p software (Pachitariu et al., 2017). Images from all blocks (task blocks and one passive block) within a day were registered together. For the chronic imaging dataset, identical cells were matched across days by using the Matlab-written software CellReg (Sheintuch et al., 2017). For calculating ΔF/F_0_ of calcium signals, F_0_ was defined in the following way for each cell: after taking a chunk of imaging frames (2000 frames, corresponding to ~2.5 min) every 500 frames, an empirical histogram was estimated by using a diffusion kernel density estimator (Botev et al., 2010) with a Matlab function kde.m (Botev, 2022) and the mode of the histogram was defined as the F_0_ value within each chunk. The F_0_ trace was then estimated by spline interpolation using the mode values of each chunk.

#### RF mapping

First, the position of sparse noise stimuli at each imaging frame was spatially compensated against eye movements by using the deviation angles of the pupil relative to its median location. Any imaging frames in which the deviation angles were greater than ten degrees were excluded from analysis. Second, the imaging frames corresponding to the sparse noise onsets were defined and the median onset responses were calculated for each stimulus (both black and white stimuli at all 14 × 22 locations). Third, we determined if a cell had significant ON and OFF subfields by comparing the median onset responses to the white and black sparse noises, respectively, across the visual field (rank sum test followed by Sidak correction). The sparse noise locations with *P* < 0.05 were defined as the RFs. Only those cells that exhibited positive responses to the receptive field stimuli were analyzed. For cells with single ON or OFF subfields, the 2D Gaussian function was fitted on the RF after subtracting the mean onset responses outside of RFs and the resulting two sigma ellipse was used to calculate the distance to the BR and TL locations. The Matlab function D2GaussFunctionRot.m was used for fitting a 2D Gaussian function (Nootz, 2022).

#### Attentional modulation indices

Three different types of attentional modulation indices were calculated in this study 1) for assessing spatial attention to BR and TL locations separately (Figure 3), 2) for assessing the reliability of attentional modulation within the same blocks in a session (Figure 4G), and 3) for assessing the reliability of attentional modulation between the first and third blocks in the same sessions (Figure 4H).

1) Responses to all sparse noise stimuli within RFs were averaged after subtracting the median ΔF/F_0_ signal during the baseline period (~470 ms before the onsets) for each repetition. The response peak was defined by using the sparse noise stimulus that induced the maximum response (i.e., RF center). The ΔF/F_0_ values around the peak (from 1 frame before the peak to 5 frames after the peak, corresponding to 0.5 s) were summed for each block type to get R_BR_ and R_TL_, and the attentional modulation index was calculated as follows:

(a) $$\;Atten.\;Mod.\;Ind.\;=\frac{R_{BR}-R_{TL}}{R_{BR}+R_{TL}}$$

This index is for assessing attentional modulation in BR blocks relative to TL blocks. The sign was reversed when assessing attentional modulation in TL blocks relative to BR blocks (Figures 3G and 3H). The significance of attentional modulation was examined by bootstrap test: we first sampled the summed ΔF/F_0_ values around the peak with replacement for all RF sparse noise stimuli given during the corresponding blocks for 10,000 times, and then calculated the mean for each of the bootstrap replicates. *P*-values were calculated as the percentage of the replicates that violated the observed relationship between BR and TL blocks.

To assess spatial attention to BR and TL locations collectively for the neurons whose RFs overlapped with either location, responses of neurons when mice attended in (R_in_) and out of (R_out_) their RF locations were calculated in the same way as R_BR_ and R_TL_, and the modulation index was calculated as follows:

(b) $$\;Atten.\;Mod.\;Ind.\;=\frac{R_{in\;}-R_{out\;}}{R_{in\;}+R_{out\;}}$$

This equation was used for calculating the following two attentional modulation indices.

2) To assess how stable attentional modulation was within a block, a block was separated into halves such that each sparse noise stimulus had the same number of onsets. Spatial attention within the first and third blocks were assessed by using the equation (*b*). The mean responses in each half of the blocks were used as R_in_. To control for any time-dependent changes in sparse noise responses, the second blocks were also separated into halves, the first half was used as R_out_ when calculating the indices for the two halves of the first blocks, and the second half was used for the two halves of the third blocks.

3) To assess how stable attentional modulation was between separate blocks within a session, we compared attentional modulation between the first and third blocks by using the equation (*b*). R_in_ and R_out_ were defined in the following way: R_in_ for the first and third blocks were defined as the mean sparse noise responses during the second half of the first blocks and the first half of the third blocks, respectively; and to control for any time-dependent changes in visual responses, R_out_ for the first and third blocks were defined as the mean sparse noise responses during the first and second half of the second blocks, respectively.

#### Running modulation index

A session was divided into running (running speed ≥ 1 cm/s) and stationary (running speed < 1 cm/s) epochs, and for each epoch the responses to all sparse noise stimuli within RFs were averaged after subtracting the median ΔF/F_0_ signal during the baseline period. The ΔF/F_0_ values around the peak (from 1 frame before the peak to 5 frames after the peak, corresponding to 0.5 s) were summed for running and stationary epochs separately to get R_Run_. and R_Stat._, respectively, and the running modulation index was calculated as follows:

$$\;Running.\;Mod.\;Ind\;=\frac{R_{Run.\;}-R_{Stat.\;}}{R_{Run.\;}+R_{Stat.\;}}$$

The within-block and between-block reliability of running-dependent modulation was assessed by separating blocks in similar ways. The significance of running-dependent modulation was examined in the same way as attentional modulation.

#### Running-onset activity

Onset of running was defined as the time point at which mice started running after more than 1 second of stationary period. For each running onset, the ΔF/F_0_ signal were averaged over the next 10 imaging frames (~ 0.8 s) after subtracting the median ΔF/F_0_ signal during the baseline period (10 imaging frames before running onsets), and the median of the averaged signal across all running onsets was used as the index for running-onset activity. The Wilcoxon signed-rank test was used to determine the significance.

### Analysis of *in situ* hybridization data

Cells were segmented with CellPose (Stringer et al., 2021) to get the region of interest (ROI) for each cell. The ROI information was exported as text files that were readable with ImageJ, with which any incorrectly defined ROIs were removed and any missing ROIs were added manually. Then, ImageJ ROIs were loaded into Matlab (Muir, 2022) and overlap between GCaMP6- and Gad2-positive cells was analyzed. If any GCaMP6-positive cells had an overlap with Gad2-positive cells in more than 80% of their areas, we defined them as co-expressing cells. The expression of GCaMP6 was confirmed to be restricted to excitatory neurons (only 3 out of 966 GCaMP6f expressing cells were positive for the GABAergic neuron marker Gad2).

### Statistics

#### Data from multiple animals

Instead of directly averaging data pooled across animals, we performed a hierarchical bootstrap procedure (Saravanan et al., 2020) to compute confidence intervals and perform statistical tests. To perform the paired comparisons of behavioral data (Figures 1E, 1F, 1J, 1K, S1A–S1D, and S2), we first randomly sampled animals with replacement and then sampled sessions with replacement from each of the resampled animals (for 10,000 times). For each of these bootstrap replicates, the paired data was randomly shuffled before calculating the statistic of interest (e.g. mean or median of differences of the pairs). The shuffled statistic values were compared against the mean (or median) difference of the original pairs to calculate the *P*-values. Confidence intervals were computed with the bootstrap replicates (without shuffling). For the neural data (Figures 2I, 2J, 3F, 3H, 4D, 4F, S6D, S6E, and S8B), we first randomly sampled animals with replacement and then neurons of a selected mouse with replacement. The statistic of interest was then computed from each of the bootstrapped samples. The bootstrapped statistic was used for calculating the *P*-values and confidence interval. Where necessary, *P*-values were adjusted by the Benjamini-Hochberg procedure with 0.05 false discovery rate (FDR).

#### Data of individual animals

When testing for distance-dependent differences in attentional modulation in the example sessions (Figures 3E and 3G), bootstrap test was applied: we resampled neurons of each category with replacement for 10,000 times. The mean difference of the two categories was computed for each of the bootstrap replicates and compared against that of the original samples to calculate *P*-values.

#### Sub-selecting sessions

Behavioral data was obtained from 8 animals for 111 sessions in total. All sessions were analyzed in Figures 1E, 1F, and S3. When analyzing basic behavioral variables (Figure S1), four sessions in which running speed was not recorded were excluded. One additional session in which mice kept running was excluded from analysis when comparing the task performance between running and stationary periods (Figure S2). To compare the task performance and reaction time between BR and TL blocks (Figures 1J and 1K), we selected 55 sessions in which mice showed spatial attention phenotype to the similar level with the following criterion: a block was defined as ‘focally attending’ if more than 50% of catch trials had reaction times that exceeded 95 % confidence interval of reaction times of go trials in the other block type. This compares reaction times to the identical target location between different block types (the rewarding stimulus in catch trials of TL blocks was shown at the same location in go trials of BR blocks, for example). Sessions in which no focal attention blocks were defined or focal attention blocks were not defined reliably in the same block type were not included in the across-days reliability analysis. For neural data analysis, we analyzed from 4 animals which performed the task for 3 blocks (69 sessions). One mouse, which directed focal attention to the TL location, was excluded from neural data analysis, resulting in 42 sessions in total. To assess the reliability of attentional modulation across day (Figure 4), we analyzed only session in which mice showed clear spatial attention phenotypes as defined by the above criterion.

## Supplementary Material

Supplemental information can be found online at https://doi.org/10.1016/j.neuron.2022.08.028.

Supplementary Material

## Data Availability

All data reported in this paper will be shared by the lead contact upon request. Processed data of mouse behavior and calcium imaging have been deposited at Zenodo and are publicly available as of the date of publication. The DOI is listed in the key resources table. Software used for the spatial attention experiment and analysis codes have been deposited at Zenodo and are publicly available as of the date of publication. The DOI is listed in the key resources table. Any additional information required to reanalyze the data reported in this paper is available from the lead contact upon request.

## Highlights

- Mice can deploy spatial attention to different locations within a hemifield
- Spatial attention modulates neurons with receptive fields at attended location
- Individual neurons are independently modulated by spatial attention and running
- Spatial attention and running modulate single V1 neurons with distinct dynamics

## In brief

Cortical responses are influenced by cognitive and behavioral states, but how these states modulate individual neurons is unknown. Kanamori and Mrsic-Flogel show that despite similar effects on neural responses, spatial attention and running act independently to modulate individual neurons in the primary visual cortex, suggesting separable mechanisms for their implementation.
