# Supplementary material for: Independent response modulation of visual cortical neurons by attentional and behavioral states
Source: Neuron. Author manuscript; Available in PMC 2025 Nov 4. (PMC7618321; doi:10.1016/j.neuron.2022.08.028)
Supplement: Supplementary Material [file EMS209710-supplement-Supplementary_Material.zip › 1-s2.0-S0896627322008030-mmc1.pdf]

Neuron, Volume *110*

Supplemental information

Independent response modulation of visual cortical neurons by attentional and behavioral states

Takahiro Kanamori and Thomas D. Mrsic-Flogel

Supplemental information

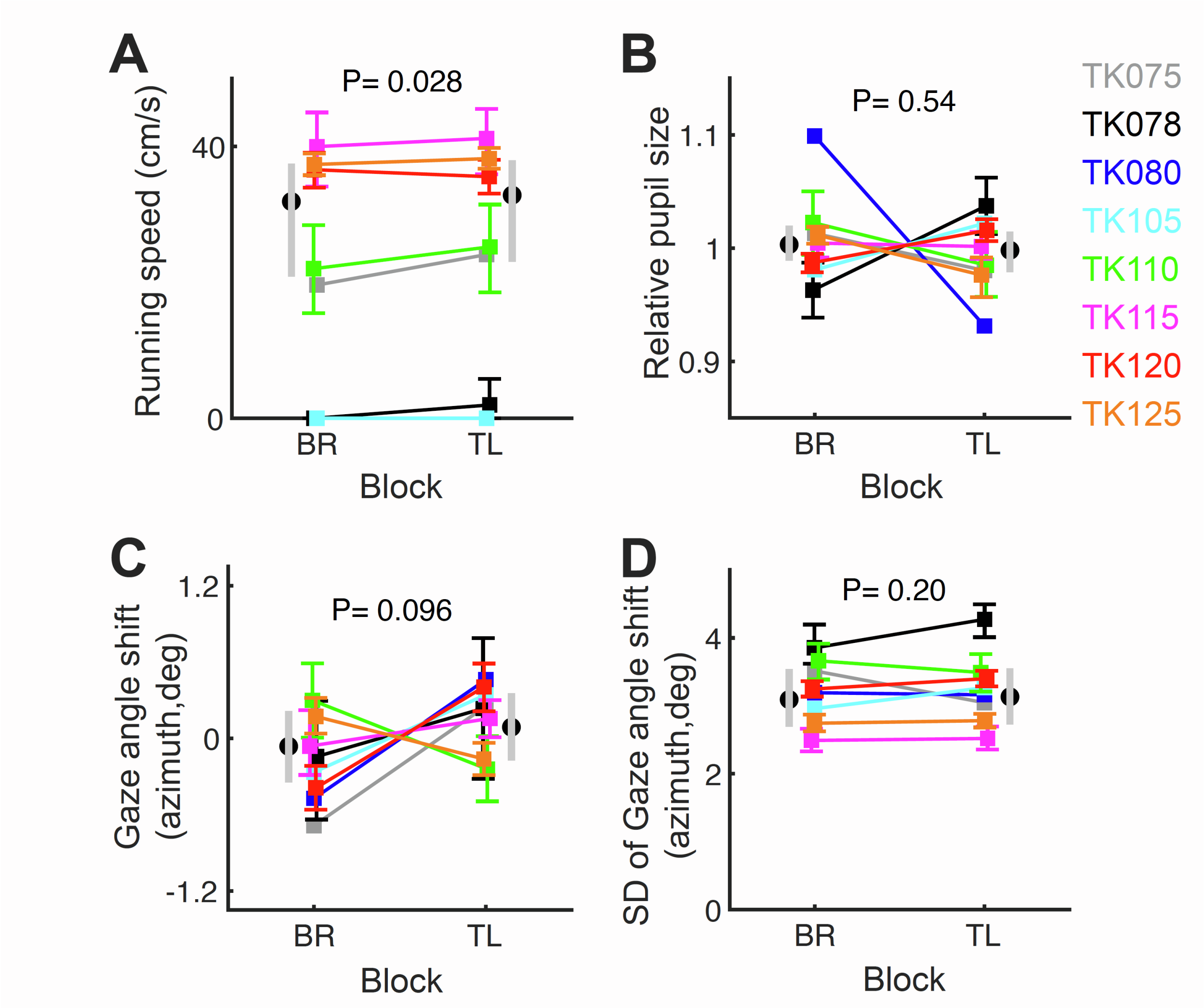

Supplemental Figure S1 | No substantial differences in running behavior, arousal level and eye movements between blocks. Related to Figure 1.

(A) Running speed.

(B) Relative pupil size (normalized by session's median value).

(C) Gaze angle shift in azimuth. Values are relative to session's median pupil location.

(D) Standard deviation of gaze angle shift. For all panels, Data from different animals are shown in different colors (Mean with 95% confidence interval of bootstrapped mean). *P*-values determined by hierarchical bootstrap. The mean values across animals are shown with black circles with 95% confidence interval of bootstrapped mean (grey lines). 107 sessions from 8 mice were analyzed. Different colors represent different animals. Data from the same animals are connected by lines.

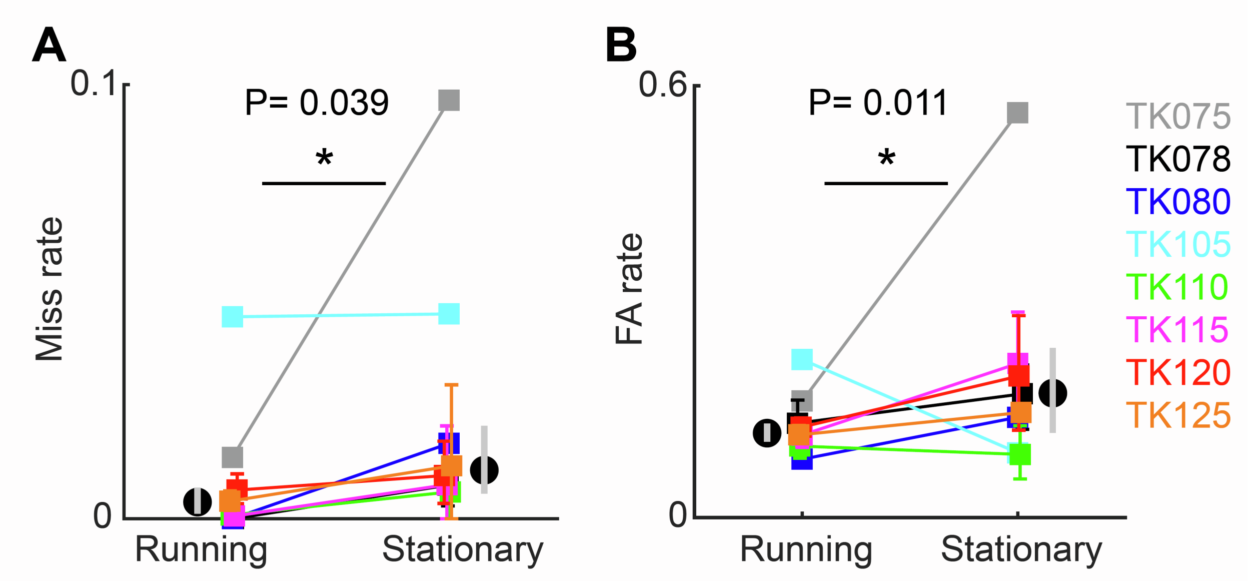

Supplemental Figure S2 | Task performance is better during running than during stationary period. Related to Figure 1

(A) Miss rate.

(B) False alarm rate.

Data from different animals are shown in different colors (Mean with 95% confidence interval of bootstrapped mean). The mean values across animals are shown with black circles with 95% confidence interval of bootstrapped mean (grey bars). *P*-values determined by hierarchical bootstrap. 106 sessions from 8 mice were analyzed.

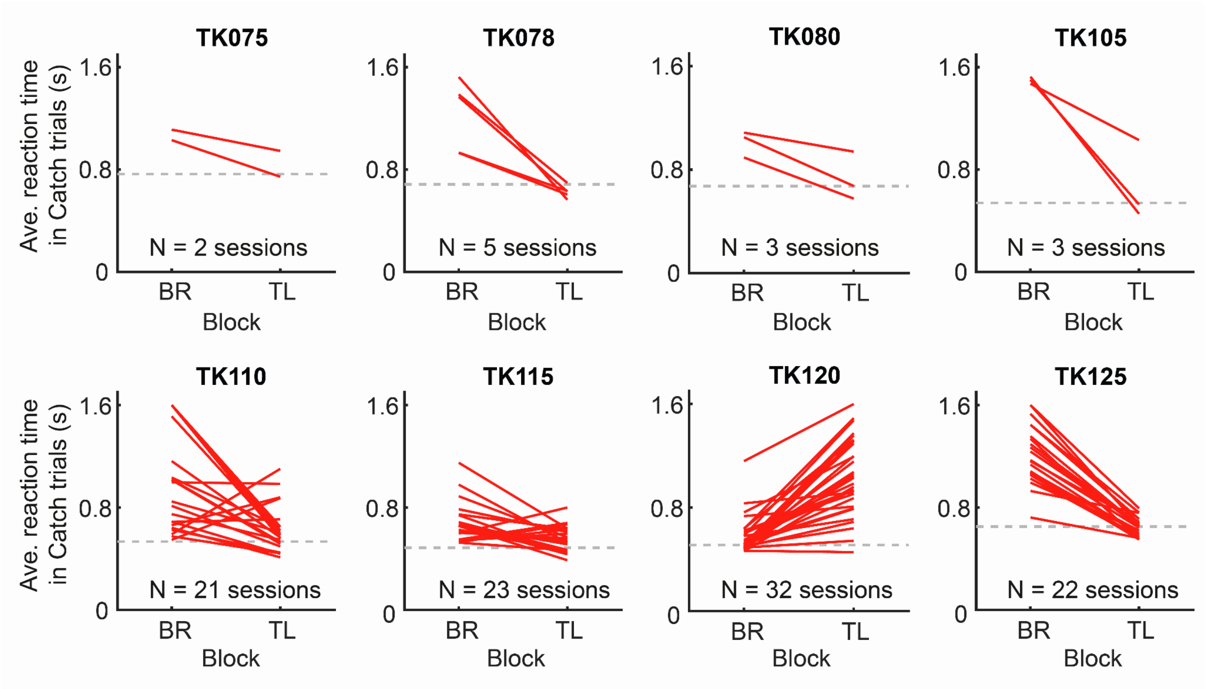

Supplemental Figure S3 | Location bias of focal attention is mostly fixed in each animal. Related to Figure 1

Average reaction time in bottom-right (BR) and top-left (TL) blocks. Dashed lines, mean reaction time in go trials.

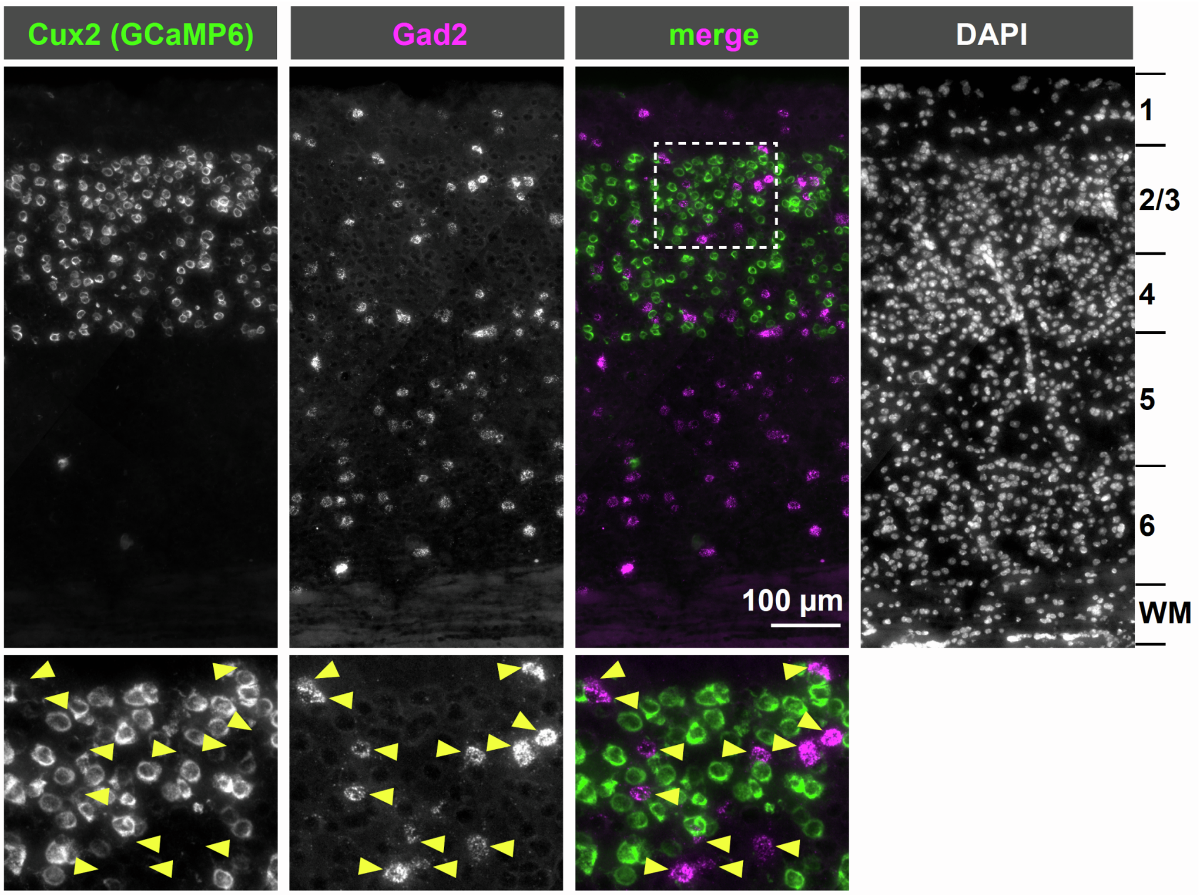

Supplemental Figure S4 | GCaMP6 expression is exclusively in excitatory neurons in the upper layers in V1. Related to Figure 2.

In situ hybridization images of a brain section in V1 from Cux2-CreERT2 / Ai148 transgenic animal. GCaMP6-positive cells rarely express the inhibitory neuron marker Gad2 (3 out of 966 cells). Yellow arrowheads indicate where Gad2-positive inhibitory neurons are.

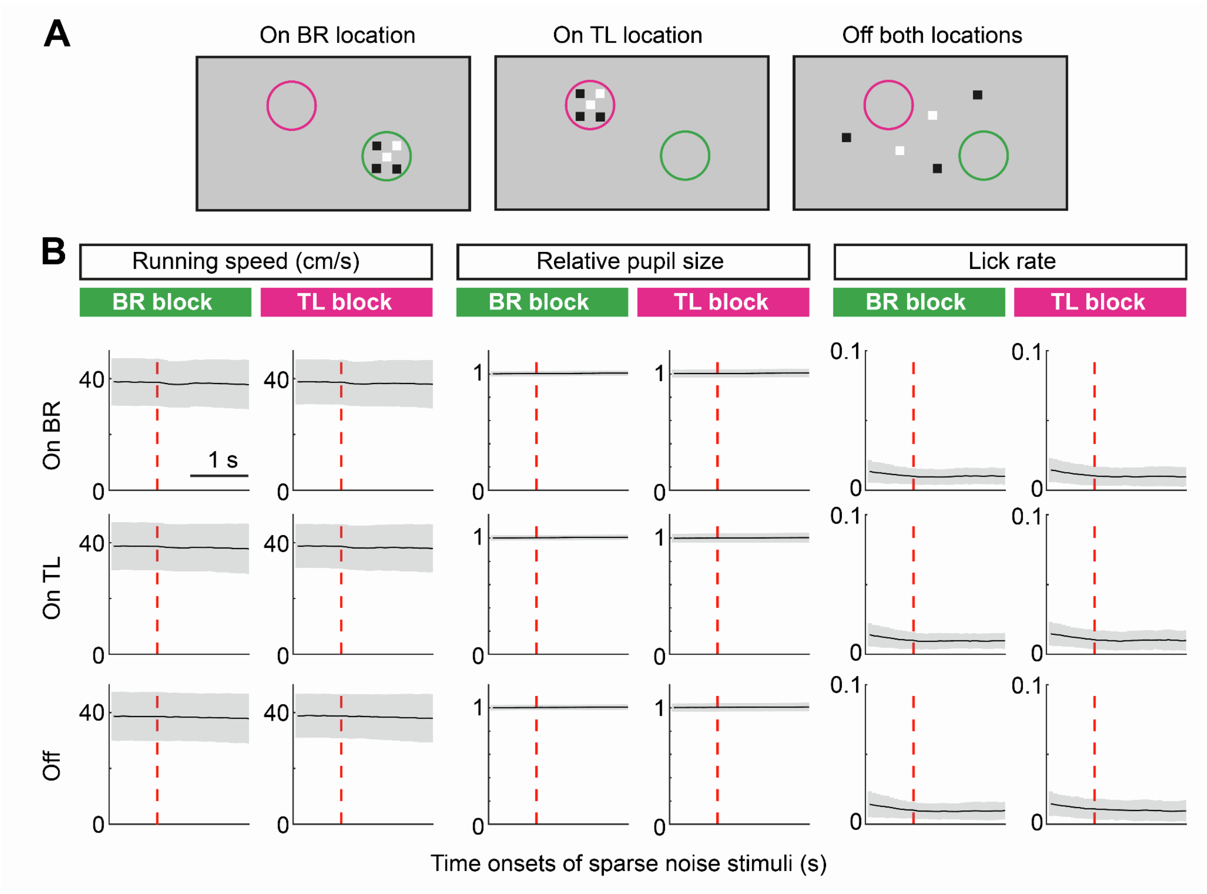

Supplemental Figure S5 | Sparse noise stimuli induce no overt behavioral responses. Related to Figure 3.

(A) Behavioral responses to 32 sparse noise stimuli (16 each for white and black) overlapping with BR location, TL location, or neither location were analyzed. For simplicity, only five stimuli are shown in each schema.

(B) Sparse noise stimuli at any location induced no responses in running speed (left column), pupil size (middle column, normalized by each session's median value), or lick rate (right column). Red dashed lines indicate the onsets of sparse noise stimuli. Shaded area, 95% confidence interval.

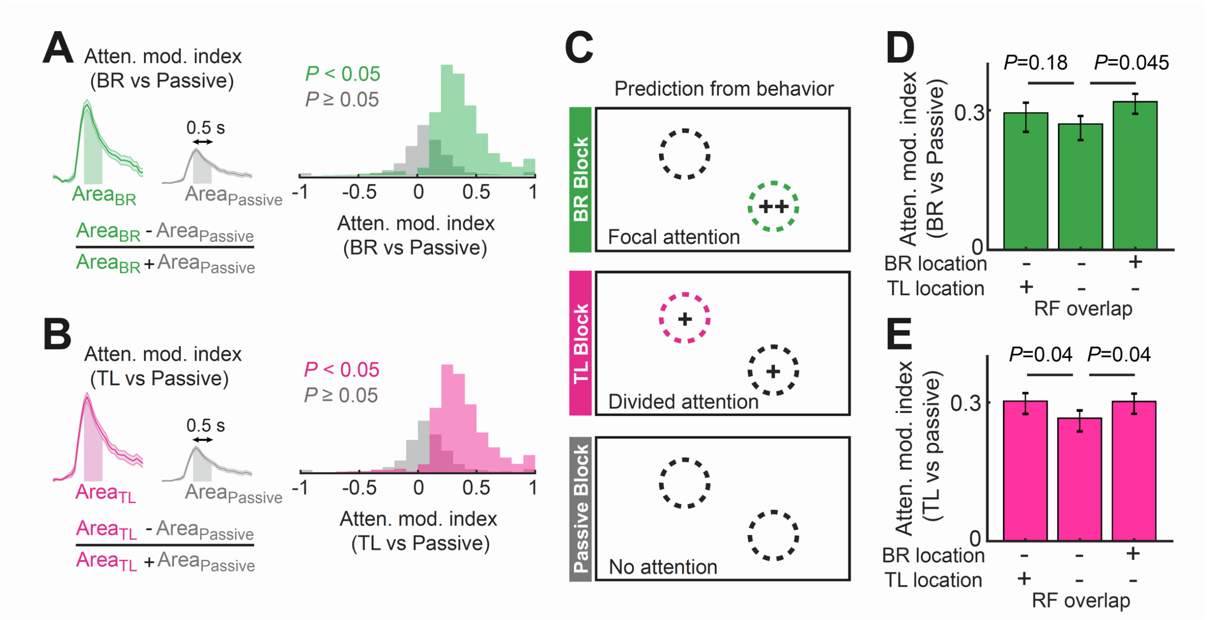

Supplemental Figure S6 | Attentional modulation of local layer 2/3 excitatory neurons relative to passive condition. Related to Figure 3.

(A) Attentional modulation index to assess attentional modulation in BR blocks relative to passive blocks. Left, attentional modulation index. Right, distribution of the attentional modulation index (green, significantly modulated; grey, not significantly modulated). n = 11,085 RFs.

(B) Attentional modulation index to assess attentional modulation in TL blocks relative to passive blocks. Left, attentional modulation index. Right, distribution of the attentional modulation index (magenta, significantly modulated; grey, not significantly modulated). n = 11,085 RFs.

(C) Schema showing where mice direct spatial attention in BR and TL blocks (plus symbols). Predicted from behavioral phenotypes.

(D) Attentional modulation in BR blocks relative to a passive block. Neurons whose RFs overlapped with BR but not TL location were more strongly modulated than those whose RFs were distant from both locations. Hierarchical bootstrap test. *P*-values were adjusted by the Benjamini-Hochberg procedure (FDR = 0.05).

(E) Attentional modulation in TL blocks relative to a passive block. Not only neurons whose RFs overlapped with BR location but also those whose RFs overlapped with TL location were more strongly modulated than those whose RFs were distant from both locations. Hierarchical bootstrap test. *P*-values were adjusted by the Benjamini-Hochberg procedure (FDR = 0.05).

Note that most neurons exhibited smaller visual responses in the passive block than in BR/TL blocks, indicating that visual responses were strongly modulated by task engagement when mice performed the task. Attentional modulation relative to passive blocks were assessed on top of the response modulation caused by task engagement.

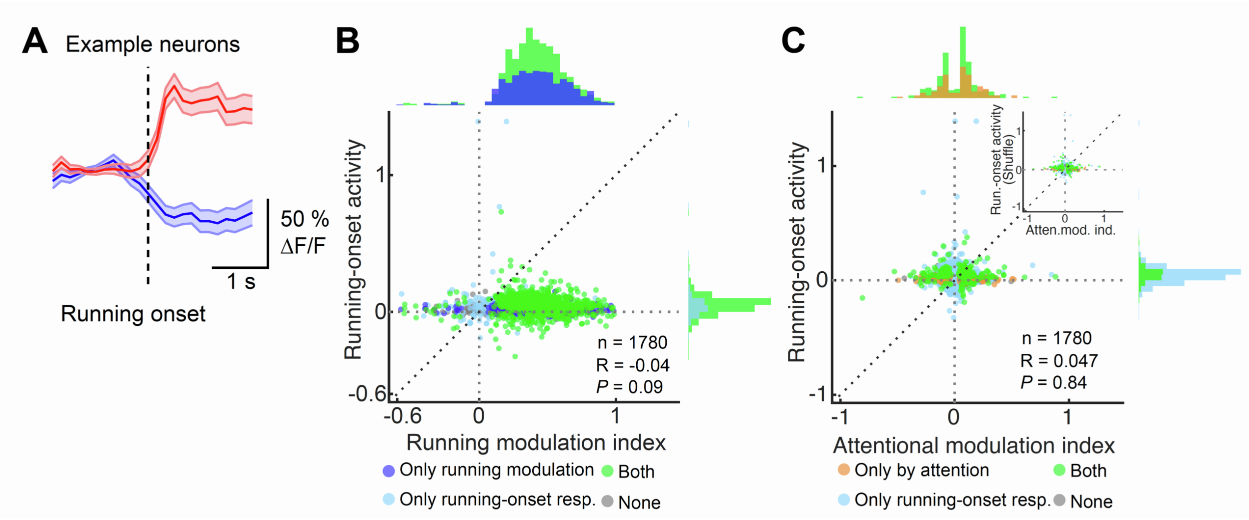

Supplemental Figure S7 | No correlation between the magnitude of running-onset activity and attentional modulation in layer 2/3 neurons. Related to Figure 4.

(A) Two example neurons that respond positively (red) and negatively (blue) at the onset of running.

(B) Independent modulation of visual response and baseline activity by running.

(C) Independent modulation of visual response by spatial attention and baseline activity by running. Inset, the same scatter plot but after shuffling the running-onset activity.

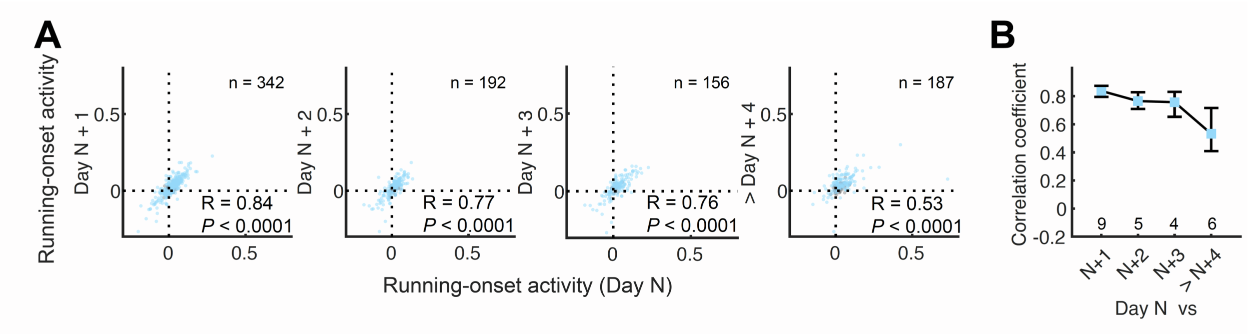

Supplemental Figure S8 | Running-onset activity in single layer 2/3 neurons is stable across days. Related to Figure 4.

(A) Scatter plots comparing running-onset activity between day N and consecutive days. Data from more than four days after the first day was pooled together.

(B) Correlation of running-onset activity across days. Error bars, 90% confidence interval of bootstrapped mean. The numbers of session pairs are indicated at the bottom.

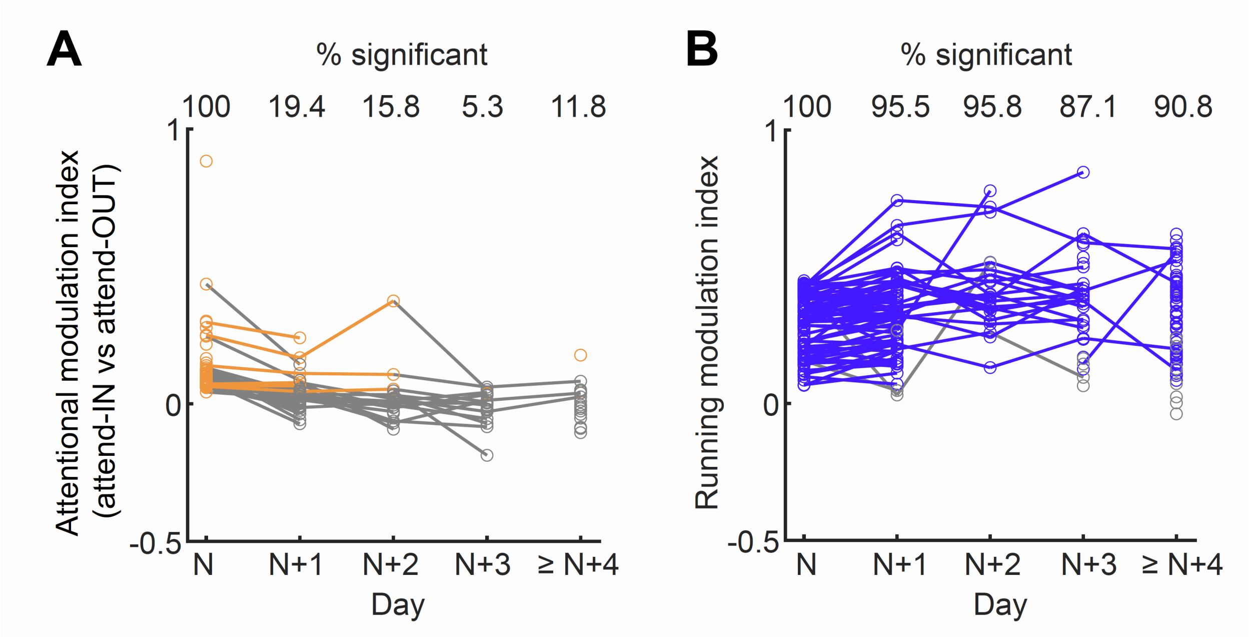

Supplemental Figure S9 | Response modulation by spatial attention is less reliable across days. Related to Figure 4.

(A) Across-day changes of attentional modulation index for neurons that were significantly positively modulated on the first day (n = 51).

(B) Across-day changes of running modulation index for neurons that were significantly positively modulated on the first day (n = 117). Neurons whose running modulation indices on day N are in the same range as the starting attentional modulation indices are sub-selected.

Colored (or gray) circles correspond to neurons with (or without) significant response modulation on each day. Individual neurons that were significantly (or not significantly) modulated on one day are connected with the values on the day before with colored (or gray) lines (circles without a connecting line are cells that do not have data on the day before). Numbers shown on top are the percentage of neurons that were significantly positively modulated on each day. Decrease in the number of data points across days comes from a smaller number of recorded sessions on consecutive days (i.e., the number of datasets on day N+3 is smaller than that on day N).

This figure shows that modulation by running was still more stable over time even for those neurons whose running modulation indices on the first day were at comparable values to the attentional modulation index.

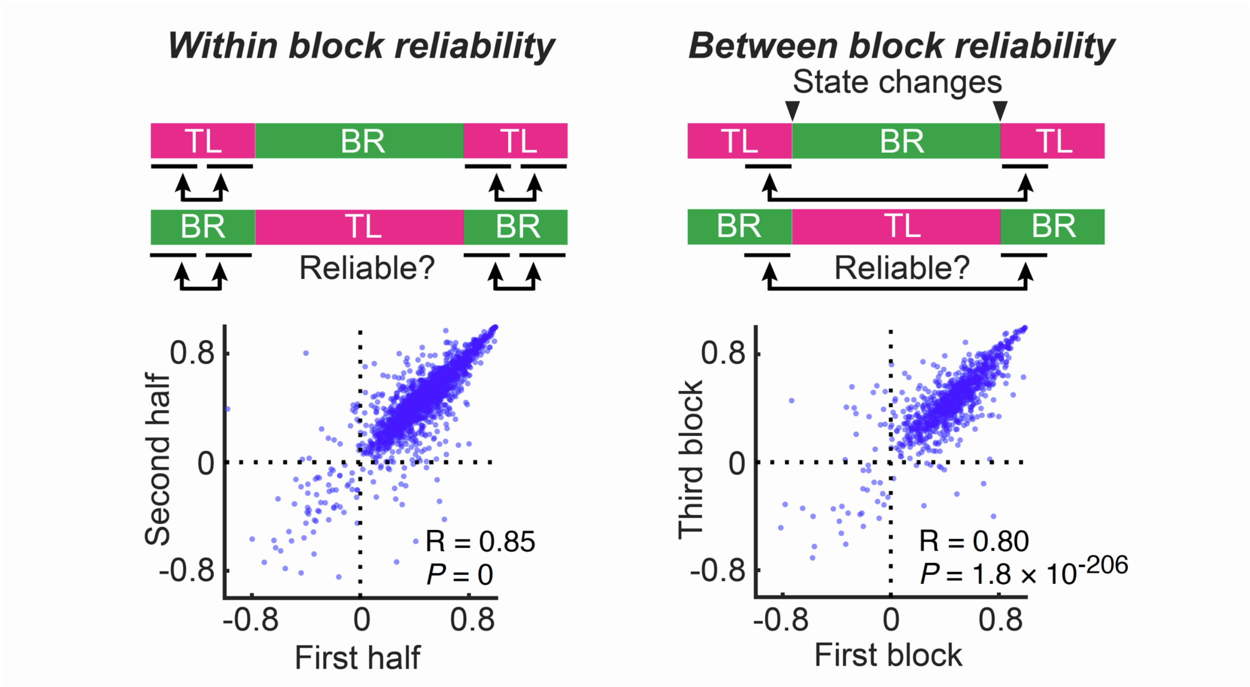

Supplemental Figure S10 | Response modulation by running is reliable within a session. Related to Figure 4.

Left, response modulation by running is reliable within blocks. Each of the first and third blocks was separated into halves (horizontal bars) and modulation indices were calculated for each half (see STAR Methods). The scatter plot compares the running modulation index between the first and second half of the same blocks. Right, response modulation by running is reliable between the first and third blocks within a session. The running modulation indices were calculated separately for the first and third blocks and compared in the scatter plot. n = 934 RFs.
